# Bone Morphogenetic Protein 15 (BMP-15) Improves In Vitro Mouse Folliculogenesis

**DOI:** 10.3390/ani13060980

**Published:** 2023-03-08

**Authors:** Jakree Jitjumnong, Pin-Chi Tang

**Affiliations:** 1 Department of Animal Science, National Chung Hsing University, Taichung 40227, Taiwan; 2 The iEGG and Animal Biotechnology Center, National Chung Hsing University, Taichung 40227, Taiwan

**Keywords:** alginate encapsulation, bone morphogenetic protein 15, estradiol, glutathione, in vitro fertilization, preantral follicle, progesterone, reactive oxygen species

## Abstract

**Simple Summary:**

Multilayered secondary follicles were encapsulated and cultured for 12 days in a 3D culture system by using alginate supplemented with bone morphogenetic protein 15 (BMP-15). Alginate is used in in vitro culture systems to mimic the natural ovarian environment by maintaining the 3D follicular architecture, cell–cell interactions, and paracrine signals underlying direct follicle development. Typically employed for follicle culture, alginate prepared at a concentration of 0.5% *w*/*v* led to considerable developmental competence and steroidogenesis. BMP-15 is a member of the superfamily of transforming growth factor-beta (TGF-β), a protein that is necessary for ovarian follicular growth. This protein functions primarily by attaching to its receptor on the surface of granulosa cells, which are also essential for the development of ovarian follicles. Regarding observations of follicle growth, in vitro culture using 0.5% alginate supplemented with 15 ng/mL BMP-15 can promote the production of mature oocytes and increase steroidogenesis. However, mature oocytes retrieved in vitro were subject to hypoxia conditions.

**Abstract:**

Multilayered secondary follicles were encapsulated in a 0.5% alginate matrix and cultured in a 3D culture system supplemented with bone morphogenetic protein 15 (BMP-15; 15 ng/mL) for 12 days. The in vitro development of ovarian follicles was evaluated. On day 12, the follicle diameter, follicle survival rate, and antrum formation rate were significantly higher for follicles cultured in BMP-15-supplemented medium than those cultured in regular medium. The percentage of ovulated metaphase II oocytes retrieved from follicles cultured in BMP-15-supplemented medium was greater than that of oocytes retrieved from follicles cultured in regular medium. The secretion of P4 was significantly higher on days 6, 8, and 10 in follicles cultured in BMP-15-supplemented medium. The result for E2 tended toward significance on day 12. Intracellular reactive oxygen species levels were higher and glutathione levels were lower in mature oocytes from the in vitro culture than in mature oocytes from an in vivo control. A 3D culture system using an alginate matrix and supplemented with BMP-15 effectively improves the outcomes of in vitro ovarian follicle culture.

## 1. Introduction

In vitro ovarian follicle cultivation systems have enabled the development of fertility preservation methods for patients with cancer [1]. Cancer treatments, including chemotherapy and radiotherapy, can cause infertility in girls and women. These treatments reduce the follicle pool and cause early ovarian failure. Patients with cancer can choose to freeze their ovarian tissue prior to treatment; however, autotransplant of cryopreserved ovarian tissue following cancer treatment poses the risk of reintroducing cancer cells [2]. In vitro follicle cultures can be used with cryopreserved ovarian tissue to produce mature eggs and, thereafter, embryos [3]. Isolated and in vitro cultivation of ovarian follicles have been investigated as an alternative approach to overcome the limitations of ovarian tissue transplantation [4]. In vitro production of isolated immature ovarian follicles is a promising option for fertility preservation in adult or prepubertal patients with cancers that may migrate to the ovaries [5]. In this regard, 2-dimensional (2D) and 3-dimensional (3D) culture systems have been developed. These methods enable the development of immature follicles in vitro, resulting in the production of oocytes that can develop, be fertilized, and give rise to live birth in mice [6].

Due to the lack of correlation between the cultivated follicle microenvironment and in vivo environments, unlike the 3D system, the 2D system does not sustain follicle integrity [7]. The 3D system has attracted more attention in recent years for follicle culture than has the 2D system. The 3D system has also been used successfully in a wider range of species [8]. Maintaining the spherical form of ovarian follicles and improving cell viability and growth in in vitro culture are the two major benefits of 3D follicle cultures [9]. Furthermore, alginate hydrogels are a common tissue engineering scaffold that replicate the ovaries by providing an ideal 3D environment and encouraging somatic cell–egg interactions to improve oocyte development [6]. Alginate is a polysaccharide polymer generated from algae that has been used in several tissue growth applications [10]. Preantral mouse, nonhuman primate, and human follicles have been grown in in vitro systems based on alginate, with mice follicles found to produce oocytes capable of fertilization and live birth [11]. Additionally, these methods have made it easier to study the mechanics, extracellular matrix, and gene expression during folliculogenesis [12]. In the present study, an in vitro 3D ovarian follicle culture was employed to maintain immature mouse follicles.

Bone morphogenetic protein 15 (BMP-15) is a member of the transforming growth factor-beta (TGF-β) superfamily. This oocyte growth factor is essential for the first phases of follicle development. Oocytes regulate the differentiation and activity of granulosa cells (GCs) and their effect on gene expression patterns in follicular somatic cells owing to BMP-15, which is critical for differentiating GCs and controlling the essential functions of GCs [13]. Oocyte-derived growth and differentiation factors may simulate how the oocyte acts on GCs and cumulus cells (CCs) to improve oocyte developmental competence under in vitro conditions [14]. An in vitro 3D ovarian follicle culture was supplemented with BMP-15 and examined with regard to the function of BMP-15 in the growth of the in vitro ovarian follicle. Although in vitro 3D ovarian follicle culture with BMP-15 supplementation has been developed to increase the effectiveness of in vitro ovarian follicle culture, there is a lack of consistency in the developmental competence of mature oocytes.

Under in vitro conditions, reactive oxygen species (ROS) are produced in oocytes when the mitochondria use oxygen to produce energy, and the level of ROS increases in vitro. In addition, ROS have the possibility of damaging oocytes, mediating oxidative stress, and complicating female reproduction [15]. Under in vivo conditions, the intracellular antioxidant defense system may maintain redox homeostasis by scavenging excess ROS [16]. Consequently, oocyte development is dependent on the proper balance of ROS and antioxidants [16]. In addition to protecting somatic cells from oxidative stress, glutathione (GSH) is a key biological regulator and also helps protect mammalian gametes [17]. GSH is regarded as one of the molecular indicators to determine cytoplasmic maturation due to its crucial function in oocyte development [17].

To the best of our knowledge, although BMP-15 is not an antioxidant, no studies have investigated the effects of alginate encapsulation with BMP-15 on the physiology of ovarian follicles grown in in vitro 3D cultures following measurement of ROS and GSH levels in mature oocytes after inducing ovulation. Taken together, these observations led us to hypothesize that in vitro 3D ovarian follicle culture supplemented with BMP-15 would result in improving the outcomes of in vitro ovarian follicle culture. Therefore, the main objective of this study was to evaluate the effect of BMP-15 supplementation in the 3D architecture culture system using an alginate hydrogel matrix on mouse folliculogenesis in vitro.

## 2. Materials and Methods

### 2.1. Animals and Chemicals

This study was performed by using 14–16-day-old ICR female mice. The mice were housed in ventilated cages in a temperature-, humidity-, and light-controlled environment (12 h of light and 12 h of darkness). The mice were fed ad libitum. The study was approved by the Institutional Animal Use and Care Committee of National Chung Hsing University (IACUC 111-185). All animals were sacrificed through cervical dislocation following anesthesia with isoflurane (Panion & BF Biotech Inc., Taoyuan, Taiwan). All reagents and chemicals were purchased from Sigma-Aldrich (St. Louis, MO, USA) unless otherwise indicated.

### 2.2. Follicle Isolation from Ovarian Tissue

**Figure 1:**
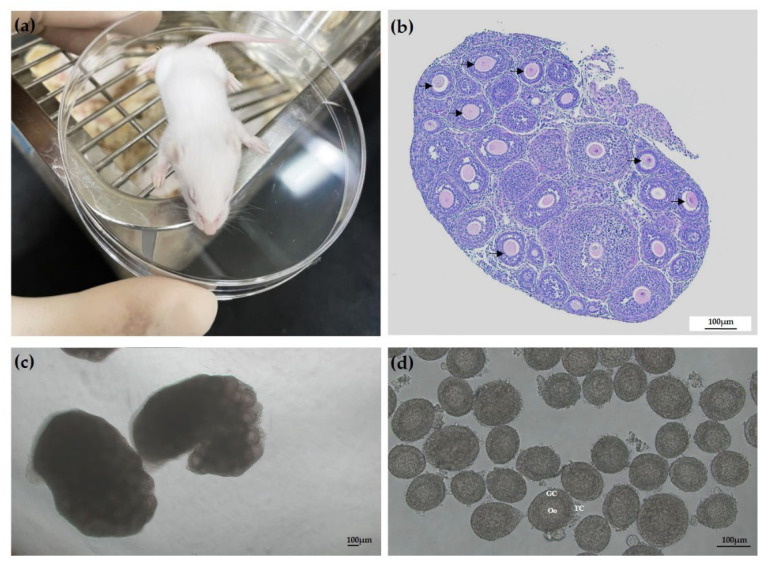
Representative images of manual isolation of single follicles from the ovaries of 14–16-day-old female mice. (**a**) Image of ICR mouse. (**b**) Hematoxylin and eosin staining of mouse ovary, which contained secondary follicles (arrows). (**c**) Representative micrographs of mice ovaries for isolation. (**d**) Secondary follicles with centrally located immature oocytes isolated from ovarian tissue. Scale bar: 100 µm. Oo: oocyte; Gc: granulosa cells; TC: thecal cells.

Ovaries from the 14–16-day-old female mice (*n* = 30) were excised; rinsed three times with phosphate-buffered saline (PBS); placed in an enzymatic medium comprising α-minimum essential medium (α-MEM; Gibco, New York, NY, USA) containing 1% fetal bovine serum (FBS; Gibco, New York, NY, USA), 0.1% type I collagenase, and 0.02% DNase I at 37 °C and 5% CO_2_ for 15 min; and then transferred to Leibovitz-15 (L-15, Gibco, New York, NY, USA) medium containing 1% FBS. Multilayered secondary follicles with a diameter of 140–180 µm were mechanically isolated under a stereomicroscope at 37 °C by using 30-gauge insulin syringes (Figure 1). Individual follicles were maintained in α-MEM containing 1% FBS at 37 °C and 5% CO_2_ for 2 h before being encapsulated. For encapsulation and culture, only follicles meeting the following criteria were selected: a diameter of 140–180 µm and a visible, immature oocyte that was round and centrally located within the follicle. Following incubation, the follicles were randomly assigned to two groups in accordance with the in vitro culture media: the CT-encapsulated follicle group, where the medium was regular culture medium, and the TM-encapsulated follicle group, where the medium was regular culture medium supplemented with 15 ng/mL BMP-15 (R&D systems, Minneapolis, MN, USA) [18].

### 2.3. 3D Culture and Alginate Encapsulation of Ovarian Follicles

To obtain alginate hydrogels, sodium alginate with a concentration of 1% (*w*/*v*) was first dissolved in deionized water. The alginate was then filtered using activated charcoal (0.5 g charcoal/g alginate) to eliminate organic impurities and increase the purity of the alginate. Subsequently, the alginate solution underwent sterile filtration using 0.22 µm filters before being reconstituted with sterile 1X PBS devoid of calcium and magnesium to produce a 0.5% (*w*/*v*) concentration. A single preantral follicle was transferred to a 5 µL droplet of alginate solution and then gently immersed in the encapsulation solution, which contained 50 mM calcium chloride and 150 mM sodium chloride, for 2 min to crosslink the alginate. The follicles in the alginate droplets were cultured in 96-well plates filled with α-MEM containing 10 mIU/mL human follicle-stimulating hormone (hFSH), 3 mg/mL bovine serum albumin, 1 mg/mL bovine fetuin, 1% insulin-transferrin-selenium (ITS 100X; Gibco, New York, NY, USA), 1% penicillin-streptomycin (P/S; Invitrogen, Carlsbad, CA, USA), and 50 µg/mL ascorbic acid; after this, they were incubated at 37 °C with 5% CO_2_ for 12 days. Every other day, half of the media (50 µL) was replaced, and conditioned media was stored at −80 °C. Follicles were considered dead if the GCs became dark and fragmented. After 12 days, the culture media was replaced by 100 µL of L-15 medium containing 10 IU/mL alginate lyase for 30 min at 37 °C. Follicles from the degraded alginate bead were removed, and the remaining alginate was completely eliminated using a new dish containing L-15 media with 1% FBS. An inverted microscope (Nikon, Tokyo, Japan) was used to capture images of encapsulated follicles on days 0, 2, 4, 6, 8, 10, and 12. The follicle diameters were measured in duplicate from the outer layers of theca cells (TCs); this was performed in Image J software (version 1.46r; National Institutes of Health, Bethesda, MD, USA).

### 2.4. Mature Oocytes Retrieved from Alginate Beads

After the follicles had been retrieved from the alginate beads, they were transferred to maturation media comprising α-MEM, 10% FBS, 3 IU/mL human chorionic gonadotropin (hCG; Biovision, Waltham, MA, USA), 10 ng/mL epidermal growth factor (EGF; R&D systems, Minneapolis, MN, USA), 10 mIU/mL hFSH, and 1% ITS at 37 °C and under 5% CO_2_ for 16–18 h. The follicles were then denuded from the surrounding CCs through treatment with 0.3% hyaluronidase, and the oocytes were gently aspirated using a glass pipette. The oocytes were washed three times with L-15 containing 1% FBS and then classified as metaphase II (MII) if a polar body was present in the perivitelline space. Degenerate oocytes were eliminated if they had fragmented or shrunk.

### 2.5. Percoll Gradient Centrifugation

Male mice were (*n* = 10) sacrificed through CO_2_ anaesthesia followed by cervical dislocation, and their epididymides were excised. Before sperm collection, fat pads around the tissues were removed. The tissues were cut with five to six incisions using microspring scissors in 1 mL of M2 medium (prewarmed to 37 °C overnight). A pipette was used to gently dispense the mouse sperm from the incisions during the 1-h incubation period. The spermatozoa were centrifuged for 2 min at 700× *g*. The sperm pellet was resuspended in M2 medium. Dulbecco’s phosphate-buffered saline (DPBS; Gibco, New York, NY, USA) was used to prepare 45% (upper phase) and 90% (lower phase; *v*/*v*) solutions of Percoll (GE Healthcare Bio-Sciences AB, Uppsala, Sweden), which were then equilibrated at 37 °C in an incubator for 20 min. A 15 mL tube containing the sperm suspension was gently filled with 1 mL in two Percoll gradient steps. Immotile sperm could interface between the two Percoll layers while the gradient was centrifuged for 15 min at 1000× *g*, but motile sperm settled as a pellet. The pellet of Percoll gradient centrifuged (PGC) motile sperm was washed once in human tubal fluid (HTF) medium (Ark Resource, Kumamoto, Japan) for 2 min at 700 × *g* after the interfaced immotile sperm and 45% and 90% Percoll solutions had been removed. The HTF medium was then used to wash the pellet of PGC motile sperm once for 2 min at 700 × *g*. After the supernatant was removed, the last sperm pellet was reconstituted with HTF. The PGC sperm were capacitated in HTF media for 30 min.

### 2.6. In Vitro Fertilization

After being retrieved, the MII oocytes were washed and incubated in 80 µL HTF medium droplets at 37 °C in 5% CO_2_ in a humidified incubator. In vitro fertilization (IVF) was conducted by transferring the MII oocytes into a new drop of HTF medium (80 µL) containing 2 × 10^6^ sperm/mL and incubating them under paraffin oil for 6 h at 37 °C in 5% CO_2_ in a humidified incubator. The oocytes were placed in a new 80 µL droplet of HTF medium, covered with paraffin oil, and cultured for 18 h in HTF medium under the same conditions. After 18 h of incubation, the number of two-cell embryos was recorded as the fertilization rate, and the embryos were transferred to an 80 µL droplet of potassium simplex optimized medium (KSOM; Merck Millipore, Darmstadt, Germany) for culture until the blastocyst stage.

### 2.7. Measurement of Steroid Hormones

Enzyme-linked immunosorbent assay kits were used to measure the concentrations of progesterone (P4; Cayman Chemical, Michigan, USA; Cat no. 582601) and estradiol (E2; Calbiotech, California, USA; Cat no. ES380S) in conditioned medium in accordance with the manufacturer’s instructions. Conditioned media were collected on days 2, 4, 6, 8, 10, and 12 of in vitro culture for both CT-encapsulated and TM-encapsulated follicles to determine the concentration of steroid hormones. The sensitivity of the P4 and E2 assays was 10.0 and 8.7 pg/mL, respectively. The intra-assay coefficients of variation were 8.75% and 12.98%, respectively. For each time point, media obtained from follicles under similar alginate conditions were pooled to ensure there were at least five samples pooled per measurement. Three independent measurements for each hormone at each time point were analyzed.

### 2.8. Measurement of Reactive Oxygen Species and Glutathione Levels in Matured Oocytes

To assess the quality of the matured oocytes, the appearance of the first polar body was used to select groups of matured oocytes from all experimental groups, and the intracellular ROS and GSH levels of these oocytes were then determined. Briefly, 2′,7′- dichlorodihydrofluorescein diacetate (H_2_DCFDA; Invitrogen, Waltham, MA, USA) and 4-chloromethyl-6.8-difluoro-7-hydroxycoumarin (CMF_2_HC; Invitrogen, Waltham, MA, USA) were used to detect intracellular ROS (green fluorescence) and GSH (blue fluorescence) levels. The mature oocytes retrieved from each treatment were incubated in the dark for 20 min at 37 °C in 0.1% polyvinyl alcohol (PVA)–DPBS containing 10 µM H_2_DCFDA or 10 µM CMF_2_HC. After incubation, the oocytes were rinsed with 0.1% PVA–DPBS, placed in 50 µL droplets, and examined using a fluorescence microscope (Olympus, Tokyo, Japan) with an ultraviolet filter (460 nm for ROS and 370 nm for GSH). The oocytes were placed on the focal plane, and the measuring area was adjusted to the size of the oocyte. The same microscope and photomultiplier settings were used in all experiments. ImageJ software (version 1.46r; National Institutes of Health) was employed to examine the fluorescence intensities of the oocytes in the fluorescence images. The experiment was performed three times.

### 2.9. Statistical Analysis

Data are described as the mean ± standard error of the mean. Follicle diameter and steroid hormone concentrations (P4 and E2) during in vitro culture were compared under the same culture day using Student’s *t*-test. The intracellular ROS and GSH levels in mature oocytes were analyzed through Analysis of Variance (ANOVA) using the general model procedure, followed by Duncan’s new multiple range test. The basis of the culture system with the follicle survival rate, antral follicles with antrum formation, MII oocytes, and the developmental competence of oocytes were compared using the chi-squared test. The analysis was performed by SAS (SAS Institute, Cary, NC, USA). Statistical significance was indicated by *p* < 0.05, and the tendency was indicated by 0.05 < *p* < 0.10 [19].

## 3. Results

### 3.1. Evaluation of Follicle Morphological Characteristics and Growth

**Figure 2:**
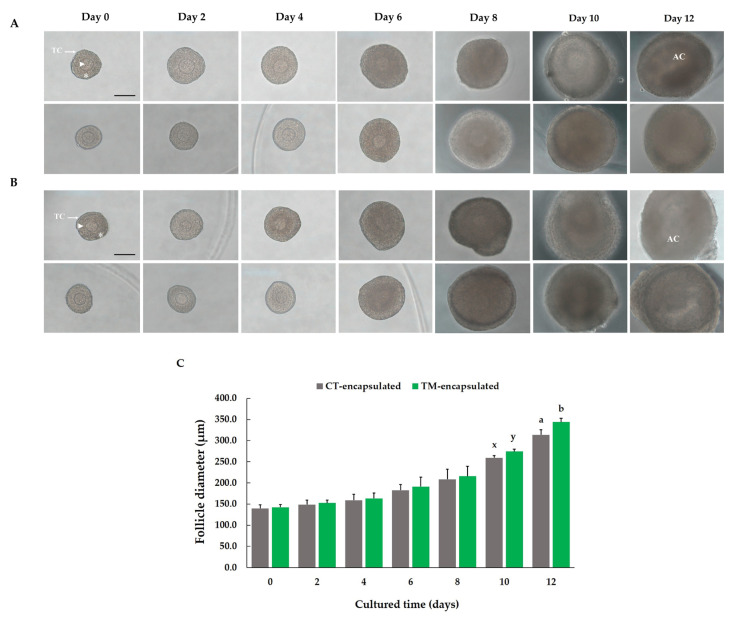
Morphology and growth of in vitro 3-dimensional culture of preantral follicles isolated from ovaries of 14–16-day-old female mice. Representative normal images of (**A**) CT-encapsulated follicles and (**B**) TM-encapsulated follicles on days 0, 2, 4, 6, 8, 10, and 12 of culture in a 3D alginate matrix. Scale bar: 100 µm. (**C**) Diameters of follicles grown in the two culture media. Within each day of cultured time (days), values with different superscript letters (a,b) indicate significant differences at *p* < 0.05, while values with different superscripts (x,y) tend to differences at 0.05 < *p* ≤ 0.10 in diameter between the two groups of follicles. Comparison between groups was performed using Student’s *t*-test. *n* = 50 follicles for ten replications. Arrowhead: oocyte; asterisk: GCs (granulosa cells); TC: thecal cells; AC: antrum cavity.

To examine the morphological change in and growth of antral follicles under the two culture conditions, we performed 3D culture of preantral follicles in a 0.5% alginate matrix for 12 days. Both the CT-encapsulated and TM-encapsulated follicles maintained their spheroidal shape until the end of the culture period (Figure 2A,B). On days 0, 2, 4, 6, and 8 of the culture periods, the diameters of the CT-encapsulated and TM-encapsulated follicles were the same (Figure 2C). However, on day 10, a tendency toward a significant difference in diameter between the two groups of follicles was discovered (259.5 ± 5.06 vs. 274.41 ± 4.98 µm, respectively; *p* = 0.056; Figure 2C). The diameter of the TM-encapsulated follicles was significantly greater than that of the CT-encapsulated follicles at the end of the culture period (343.93 ± 8.59 vs. 313.6 ± 12.20 µm, respectively; *p* < 0.05; Figure 2C).

### 3.2. Follicle Degradation and Survival and Antrum Formation

**Figure 3:**
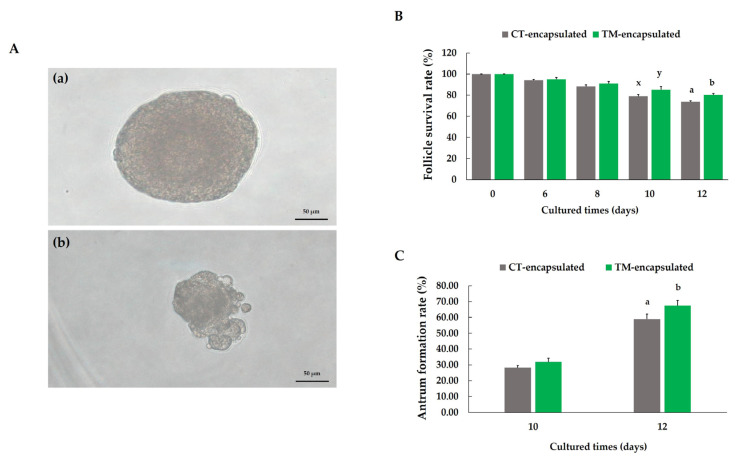
Effect of culture medium on mouse follicle growth. (**A**) Representative images of follicles without antrum formation at the end of culture period (a) and degenerated follicle (b). Scale bar: 50 µm. (**B**) Follicle survival rate. (**C**) Antrum formation rate on days 10 and 12. Different letters (a,b) indicate significant differences at *p* < 0.05, while different superscripts (x,y) tend to differ at 0.05 < *p* ≤ 0.10 in effects between two groups of follicles. Comparison between groups was performed using chi-squared test. *n* = 500 preantral follicles (grey bars) and *n* = 498 preantral follicles (green bars) for ten replications.

In two investigations, preantral follicles were isolated and first cultivated with the goal of promoting the development of antral follicles. Morphological images of antral follicles without antrum formation during in vitro culture and degenerated follicles at the end of the culture period are presented in Figure 3A. However, on the 10th day, a tendency toward a significant difference was discovered in the follicle survival rate between the CT-encapsulated and TM-encapsulated follicle groups (79.04% ± 1.45% vs. 85.19% ± 3.01%, respectively; *p* = 0.098; Figure 3B), i.e., there was a tendency toward a higher follicle survival rate in the TM-encapsulated follicles. On the 12th day, a significantly higher (*p* < 0.05) follicle survival rate was found in the TM-encapsulated follicles than in the CT-encapsulated follicles (80.19% ± 1.43% vs. 73.63% ± 1.10%; Figure 3B). After 10 days of culture, some antrum formation was observed in both cultures (Figure 3C); however, no significant difference (*p* > 0.05) in the percentage of antrum formation was discovered between the two groups of follicles (Figure 3C). The antrum formation rate was significantly higher (*p* < 0.05) in the TM-encapsulated follicles than in the CT-encapsulated follicles on the 12th day of culture (67.35% ± 3.43% vs. 58.90% ± 3.22%; Figure 3C).

### 3.3. Fertilization and Embryo Development

**Figure 4:**
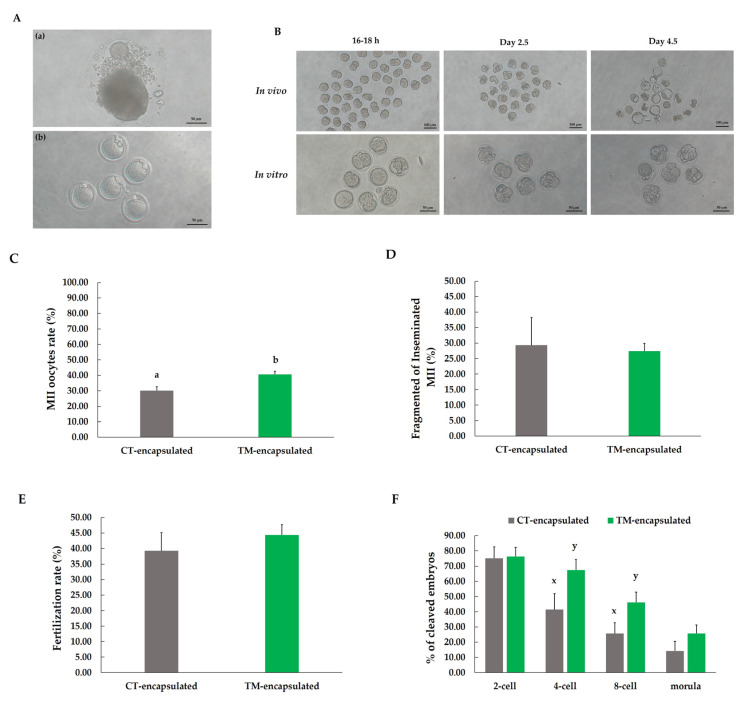
Meiotic and fertilization competence of oocytes from follicles cultured for 12 days in alginate matrix. (**A**) Representative images of follicle after 16–18 h of hCG stimulation (a) and MII oocytes (b). (**B**) Comparison of fertilization and embryo development between in vitro and in vivo mature oocytes. (**C**) Percentage of MII oocytes fertilized by motile sperm obtained through Percoll gradient centrifugation. (**D**) Percentage of fragmented inseminated MII oocytes after IVF. (**E**) Fertilization rate. (**F**) Percentage of cleaved embryo development in different stages. Different letters (a,b) indicate significant differences at *p* < 0.05, while different superscripts (x,y) tend to differ at 0.05 < *p* ≤ 0.10 in effects between two groups of follicles. Comparison between groups was performed using chi-squared test. *n* = 217 antral follicles (grey bars) and *n* = 280 antral follicles (green bars) for ten replications.

After the follicles were retrieved from the alginate hydrogel matrix on day 12, exogenous hCG was used to stimulate in vitro maturation. Images of the MII oocytes after 16–18 h of stimulation are presented in Figure 4A. The percentage of MII oocytes retrieved from follicles was significantly greater (*p* < 0.01) in the TM-encapsulated follicle group than in the CT-encapsulated follicle group (40.65% ± 2.07% vs. 30.22% ± 2.44%; Figure 4C). In addition, to analyze the developmental competence of oocytes, only mature oocytes retrieved from encapsulated follicles were fertilized using PGC sperm. Fertilization and embryo development for in vivo control compared with in vitro culture is shown in Figure 4B. The fragmentation of inseminated MII oocytes and the fertilization rate did not differ (*p* > 0.05) between the two groups of follicles after IVF (Figure 4D,E). However, the percentage of cleaved embryos tended to be higher in the TM-encapsulated follicle than in the CT-encapsulated follicle groups for 4-cell (*p* = 0.055) and 8-cell (*p* = 0.053) embryo development (67.56% ± 6.88% vs. 41.48% ± 10.40% and 46.12% ± 6.91% vs. 25.74% ± 6.96%, respectively; Figure 4F); this tendency was not found for the 2-cell and morula stages of embryo development (Figure 4F). Furthermore, no blastocysts were formed in the in vitro group in this study.

### 3.4. Steroid Hormone Production during In Vitro Culture

**Figure 5:**
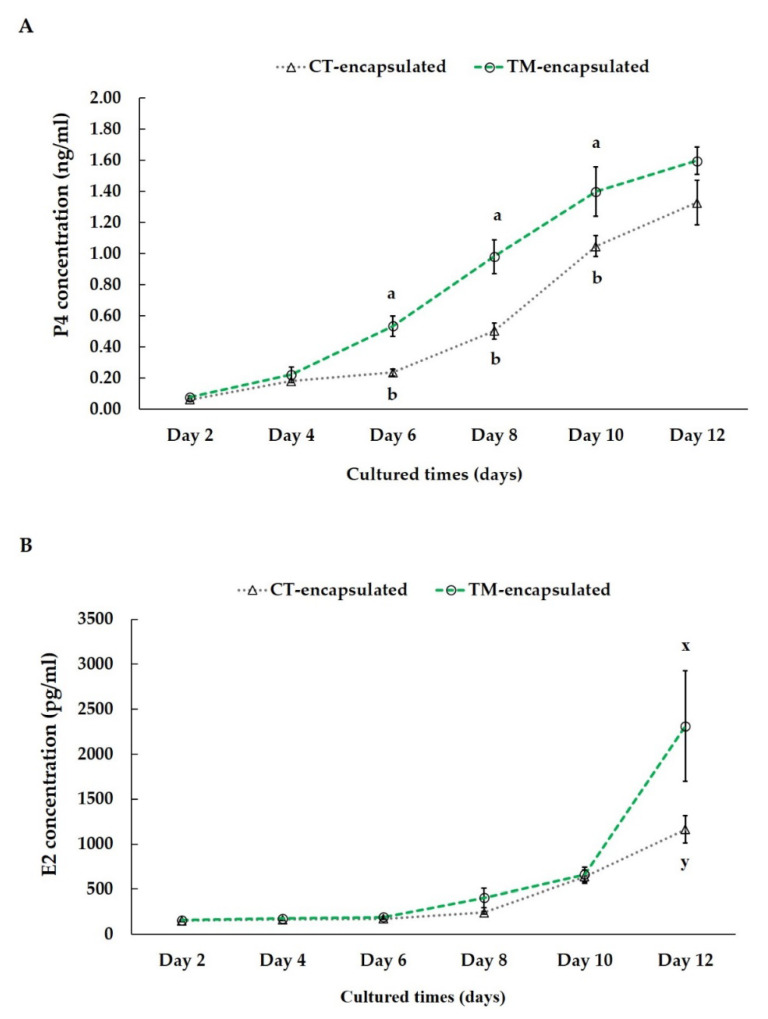
Concentrations of P4 (**A**) and E2 (**B**) in culture media on days 2, 4, 6, 8, 10, and 12 during follicle culture. Hormone concentration in the culture medium is presented on the Y-axis, and culture duration is presented on the *X*-axis. Outcomes are displayed as mean ± standard error of the mean. Different letters (a,b) indicate significant differences at *p* < 0.05, while different superscripts (x,y) tend to differ at 0.05 < *p* ≤ 0.10 between the two groups of follicles. Comparison between groups was performed using Student’s *t*-test. *n* = 3 measurements at each time point.

Steroidogenesis is crucial for maintaining oocyte development, GC differentiation, and microenvironment homeostasis [20]. Therefore, in this study, the concentrations of E2 and P4 in the culture medium during in vitro culture were detected on days 2, 4, 6, 8, 10, and 12. The P4 levels in the aliquoted culture medium revealed no significant difference between the CT-encapsulated and TM-encapsulated follicle groups until the 4th day of in vitro culture (Figure 5A). From the 6th day to the 10th day, the concentration of P4 in the medium containing the TM-encapsulated follicles was significantly higher (*p* < 0.05) than that in the medium containing the CT-encapsulated follicles (day 6: 0.54 ± 0.07 vs. 0.24 ± 0.02 ng/mL; day 8: 0.98 ± 0.11 vs. 0.50 ± 0.05 ng/mL; day 10: 1.40 ± 0.16 vs. 1.05 ± 0.07 ng/mL, respectively; Figure 5A). However, at the end of the culture period, no significant difference (*p* > 0.05) between the CT-encapsulated and TM-encapsulated follicle groups was observed. In terms of the E2 level, no significant difference was observed until the 10th day (Figure 5B). The difference in E2 levels tended to be higher (*p* = 0.070) in the group of TM-encapsulated follicles than CT-encapsulated follicles at the end of the culture period (2311.92 ± 614.96 vs. 1164.53 ± 153.86 pg/mL, respectively; Figure 5B).

### 3.5. Determination of Intracellular ROS and GSH Level of Mature Oocytes

**Figure 6:**
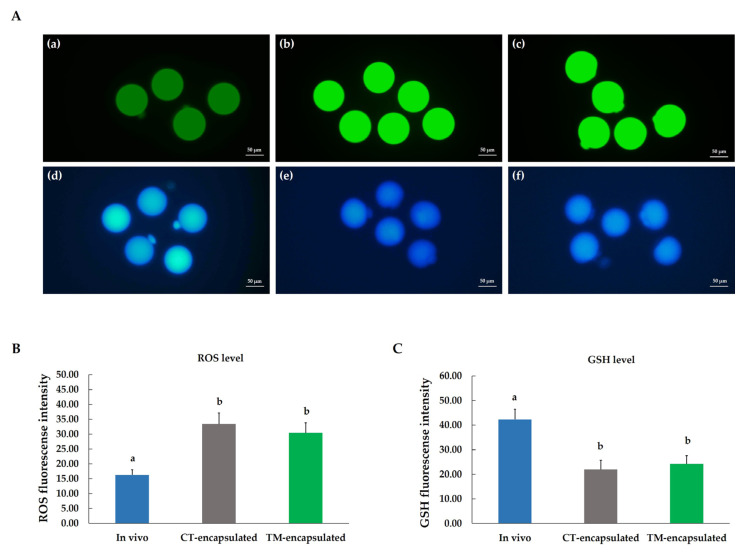
Fluorescent microscope photographs of mature oocytes collected from in vitro follicle cultures. (**A**) Oocytes were stained with H_2_DCFDA (a–c) and CMF_2_HC (d–f) to detect intracellular ROS and GSH levels, respectively. Mature oocytes retrieved from in vivo (a,d), CT-encapsulated (b,e), and TM-encapsulated (c,f) follicles. (**B**) Fluorescence intensity of intracellular ROS level. (**C**) Fluorescence intensity of intracellular GSH level. Scale bar: 50 µm. Different letters (a,b) indicate the significant differences at *p* < 0.05 in mature oocytes derived from in vivo and in vitro. Comparison between groups was performed using ANOVA followed by Duncan’s new multiple range test. *n* = 3 measurements in each group.

To determine the influence of BMP-15 supplementation in the culture medium during an in vitro 3D culture, the intracellular ROS and GSH levels in mature oocytes were measured after hCG induction for 16–18 h; the mature oocytes derived from in vivo were used as a positive control (Figure 6A). Our results demonstrated that the intracellular ROS level in mature oocytes was significantly higher (*p* < 0.05) for both the CT-encapsulated and TM-encapsulated follicle groups in comparison with mature oocytes derived from the in vivo control (fluorescence intensity: 33.45 ± 3.73 and 30.48 ± 3.38 vs. 16.34 ± 1.68, respectively; Figure 6B). Because GSH is a potent antioxidant, we also detected intracellular GSH levels in mature oocytes. These levels were significantly lower (*p* < 0.05) in both the CT-encapsulated and TM-encapsulated follicle groups compared with those derived from the in vivo control (fluorescence intensity: 21.94 ± 3.81 and 24.17 ± 3.40 vs. 42.26 ± 4.27, respectively; Figure 6C). No significant difference in those parameters was discovered between the mature oocytes from the CT-encapsulated and TM-encapsulated follicle groups (Figure 6B,C).

## 4. Discussion

Although several studies have attempted to develop in vitro 3D follicle culture systems, the understanding of in vitro follicle growth and the requirements for optimal culture conditions—such as medium composition, in vitro culture types, and techniques—are still lacking [4]. Techniques that incorporate 3D cultures are able to preserve the gap junction between cells and the follicular structure. We thoroughly investigated the effects of 3D culture by using an alginate matrix on whole follicles (CT-encapsulated and TM-encapsulated follicles) with regard to follicle development, meiotic competence, and steroidogenesis in in vitro culture. Moreover, we assessed the accumulation of intracellular ROS and GSH in mature oocytes that were retrieved from in vitro culture and compared the results with those of an in vivo control. A nonspherical, 2D in vitro culture technique has been used in previous studies to characterize the formation of preantral follicles. A 48–54% follicle survival rate and a 38–54% antrum formation rate were reported for culturing in a typical 2D culture system [21,22,23]. In the present study, we used an alginate matrix with CT-encapsulated follicles in a 3D follicle culture system and obtained a follicle survival rate of 73.63% and an antrum formation rate of 58.90%. With TM-encapsulated follicles, we observed even greater rates of follicle survival (80.19%) and antrum formation (67.35%). In 2D culture, the adhesive environment results in significant rates of spontaneous follicle disruption, substantial extracellular matrix changes, and disruption of follicle morphology. By contrast, the follicular structure can be preserved in 3D gel environments due to their in vivo–like basal lamina structure, which reduces the likelihood of spontaneous rupture [24]. Compared with conventional 2D culturing, 3D culturing with an extracellular matrix offers a superior environment for the in vitro development and survival of immature mouse preantral follicles [25]. Obtaining a substantial number of follicles for in vitro maturation and IVF may be made possible by a culture approach that encourages follicle development and oocyte maturation beginning at the early follicular stage [26]. As we discovered in our review of the literature, 0.5% sodium alginate gel is the optimal concentration for the expression of steroidogenesis-related genes. In the spherical 3D culture system, the spherical structure and physiological relationships within the follicles are well preserved [27]; thus, the proper concentration of sodium alginate matrix stimulates follicle development and induces maturity of follicles as well as the production of steroid hormones [27]. Our findings also demonstrated that, compared with CT-encapsulated follicles, TM-encapsulated follicles had greater diameters and higher survival and antrum formation rates by the end of the culture period. Additionally, the potential of an oocyte to resume meiosis was higher for the TM-encapsulated follicles, and the number of antral follicles without antrum formation was smaller for the TM-encapsulated follicles. By contrast, the subsequent rate of fertilization of mature oocytes did not significantly differ between the CT-encapsulated and TM-encapsulated follicle groups. These results suggest that TM-encapsulated follicles have better morphological follicle development and oocyte meiosis resumption.

Considering follicle development and steroid hormone production during follicular growth, P4 and E2 are the major products of proliferating GCs [28]. Our results showed that TM-encapsulated follicles had higher P4 and E2 production than those from CT-encapsulated follicles. The increased levels of these steroid hormones indicate that the GCs were well-preserved and functional throughout in vitro cultivation. It is known that E2, mainly produced by the GCs of ovarian follicles, is able to promote GC proliferation and follicle development [28]. Furthermore, it was discovered that oocytes and GCs both express P4 receptors under the circumstances of P4 production, and P4 can enable oocytes to mature [29]. During the development of ovarian follicles, the secretion of these steroid hormones is typically enhanced with the development of antral follicles [30]. In addition, the previous study has shown that the supplementation of BMP-15 and FSH to an ovarian organ culture had a greater possibility of developing antral follicles, that is, increased ovary size and promotion of ovarian function by producing significant concentrations of P4 and E2 [31]. Interestingly, the follicle development in the current study showed that follicles in both groups developed most rapidly from day 6 to day 12, which is consistent with the results of P4 and E2 production. Moreover, an increase in P4 and E2 production was higher in TM-encapsulated follicles than CT-encapsulated follicles in vitro. In bovines, higher plasma P4 levels during the initial stages of ovulation benefit the resumption of oocyte meiosis and the enhancement of oocyte quality [32]. Additionally, it has been reported that the supplementation of P4 during follicle development had no effect on the development of secondary follicles [33]. These findings suggest that it is important to evaluate P4 and E2 levels throughout in vitro culture in order to monitor follicle development. The increased GC proliferation and the steroid hormone production during the in vitro culture system were observed in TM-encapsulated follicles.

From a wider viewpoint, the proportion of antral follicles and the mean surface area of the ovaries were low in the groups without either of these substances (BMP-15 and FSH) and the groups without FSH. These findings demonstrate that BMP-15 has proliferative and maturation effects on GCs that result in improved development of follicles and expansion of the size of the ovaries [31]. This finding is consistent with those of studies showing that BMP-15 stimulates the development of primordial and primary follicles in humans and goats. Additionally, the addition of BMP-15 to the culture medium stimulated growth and antrum formation and preserved the structural integrity of isolated caprine preantral follicles after 18 days of culture [34,35]. Furthermore, an in vivo study revealed a substantial correlation between increased follicular fluid GDF9 levels and the quality of oocytes and embryos [36]. The in vitro culture maturation of oocytes is enhanced by oocyte-derived factors, including BMP-15 and GDF9, which promote GC proliferation and differentiation [37]. GDF9 has been proven effective at promoting early preantral follicle growth in human ovarian cortex tissue culture and in vitro-cultured rat preantral follicles [38]. Completely sterile BMP-15 homozygous mutants inhibited follicle development in the primordial stage [39]. BMP-15 was able to prevent apoptosis of CCs after the removal of the bovine oocyte from cumulus–oocyte complexes [40]; BMP-15 also contributed to cumulus expansion by promoting the expression of EGF-like growth factors [41]. Consequently, these oocyte-derived factors, which are regulated in a manner that is particular to oocytes, are essential for the development of follicles [37]. BMP-15 and FSH treatment increased the expression of the zona pellucida (ZP3) and proliferating cell nuclear antigen (PCNA) genes. Increased ZP3 gene expression in ovaries cultivated with BMP-15 may be associated with higher oocyte quality [31]. A glycoprotein called zona pellucid covers the mammalian egg and is crucial for sperm binding and fertilization. The major sperm receptor in mice is ZP3, which is one of the three glycoproteins that make up the mouse zona pellucida (ZP1-3) [42]. A 36-kDa protein known as PCNA was shown to express itself in both the fetal and adult ovaries of several animals [43]. In rat ovaries, PCNA expression was not seen in GCs or oocytes in primordial follicles but increased when follicle expansion began [44]. Additionally, BMP-15 supplementation had no effect on the expression of the *CYP17* gene. An enzyme known as cytochrome P450c17, which is encoded by the *CYP17* gene, produces androgen precursors that turn into estrogens during ovarian steroidogenesis [45]. Presumably, in the theca interna cells, the P450 enzyme (CYP11A1) converts cholesterol into pregnenolone within the mitochondrial inner membrane [46]. According to other molecular findings, the expression of BMP receptor IB (BMPR-IB), BMP receptor type II (BMPR-II), and FSH receptor (FSH-R) was downregulated in cultured mouse ovaries. Follicular growth depends on FSH, a glycoprotein released from the pituitary gland that binds to a particular FSH receptor on the somatic cells of the ovary [47]. Adenylate cyclase or other pathways are connected to the G protein-coupled, seven-transmembrane receptor known as FSHR. BMP-15 was shown to have an inhibitory effect on the expression of FSH receptor and to block FSH activity by reducing the expression of FSHR in rat GCs [47]. GC surfaces have been shown to include BMPR-IB and type II, which are overexpressed in the biggest follicles and upregulated in advanced atretic follicles [48]. BMP-15 may inhibit the BMPR gene, preventing follicular regression and promoting follicular growth. In accordance with this assumption, BMP-15 controls a number of genes that are involved in the control of the gene balance in the survival and apoptosis of CCs [49]. These findings support the beneficial effect of BMP-15 on follicle development during in vitro culture.

In terms of ROS and GSH production, our findings revealed increased levels of intracellular ROS in mature oocytes but decreased levels of intracellular GSH compared with the in vivo control. We speculate that osmotic and thermal stress caused by prolonged in vitro culture of mouse ovarian follicles may induce a decline in intracellular GSH in MII oocytes. In biological systems, ROS are important signaling molecules for various physiological processes, including aging, cellular apoptosis, and meiotic resumption [50,51]. Furthermore, because mammalian oocytes and embryos are highly sensitive to ROS, even a slight increase in ROS levels can impair oocyte maturation and embryo development, thereby encouraging fragmentation of the embryo [52]. From the unfertilized egg to the blastocyst, a considerable reduction in intracellular GSH concentration occurs. Maintaining a high intracellular GSH concentration in unfertilized mouse oocytes is crucial for embryonic development, according to a study that revealed that high intracellular GSH levels in mouse oocytes stimulate early embryo development [53]. The in vitro culture of mouse ovarian follicles increased ROS and decreased GSH in mature oocytes due to inevitable hypoxia conditions [54]. It is known that ROS-induced damage causes cell death or aging. ROS can cause oxidation processes that could result in the degradation of biological macromolecules such as proteins, DNA, and lipids [55]. **As a result, the low developmental competence of mature oocytes retrieved from in vitro may be due to the high level of ROS, which causes the developmental defect in blastocyst formation in the group of mature oocytes retrieved from in vitro.**

Overall, based on our results, this study clearly indicates that supplementation of BMP-15 during the in vitro culture of mouse ovarian follicles could improve in vitro follicular maturation and development. The use of steroid hormones (P4 and E2) as indicators of granulosa and theca cell function in this study was consistent with the approach of other studies, and the findings revealed that the steroidogenic pathways were active and accelerated during the in vitro culture of ovarian follicles when BMP-15 was present in the culture medium.

## 5. Conclusions

Although BMP-15 supplementation in the culture medium promotes morphological changes in ovarian follicles, resumption of oocyte meiosis, and steroidogenesis, the mature oocytes retrieved from in vitro culture are subject to hypoxia conditions due to prolonged in vitro culture. A limitation of this study is the lack of comparison between different concentrations of BMP-15 supplementation in the culture medium, which may have caused significant differences in outcomes. Furthermore, additional research is required to identify which biomaterials for 3D in vitro follicle culturing using an alginate matrix can best reduce intracellular ROS and increase GSH levels in mature oocytes.

## Data Availability

The data that support the findings of this study are available from the corresponding authors upon reasonable request.
